# The neglected burden of tuberculosis disease among health workers: a decade-long cohort study in South Africa

**DOI:** 10.1186/s12879-017-2659-3

**Published:** 2017-08-07

**Authors:** Lyndsay M. O’Hara, Annalee Yassi, Muzimkhulu Zungu, Molebogeng Malotle, Elizabeth A. Bryce, Stephen J. Barker, Lincoln Darwin, J. Mark FitzGerald

**Affiliations:** 1 School of Population and Public Health, University of British Columbia, Vancouver, Canada; 2 National Institute for Occupational Health, National Health Laboratory Service, Johannesburg, South Africa; 3 School of Health Systems and Public Health, University of Pretoria, Pretoria, Gauteng South Africa; 4 Department of Pathology and Laboratory Medicine, University of British Columbia, Vancouver, Canada; 5 Division of Respiratory Medicine, University of British Columbia, Vancouver, Canada

## Abstract

**Background:**

Health workers (HWs) in resource-limited settings are at high-risk of exposure to tuberculosis (TB) at work. The aim of this study was to estimate the rate of TB disease among HWs in the Free State Province of South Africa between 2002 and 2012 and to compare demographic and clinical characteristics between HWs and the general population with TB. This study also explores the effect of occupational variables on risk of TB among HWs.

**Methods:**

Probabilistic record linkage was utilized to identify HWs who were also registered as TB patients. This historical prospective cohort study calculated incidence rate ratios (IRR) for TB disease among HWs in Free State from 2002 to 2012. Generalized linear mixed-effects regression was used to model the association between sex, race, facility type, occupation, duration of employment, and the rate of TB.

**Results:**

There were 2677 cases of TB diagnosed among HWs from 2002 to 2012 and 1280 cases were expected. The overall TB incidence rate in HWs during the study period was 1496·32 per 100,000 compared to an incidence rate of 719·37 per 100,000 in the general population during the same time period. IRR ranged from 1·14 in 2012 to 3·12 in 2005. HWs who were male, black, coloured and employed less than 20 years had higher risk of TB. Facility type and occupation were not associated with increased risk of TB when adjusted for other covariates.

**Conclusion:**

HWs in South Africa have higher rates of TB than the general population. Improved infection prevention and control measures are necessary in all high-burden TB healthcare settings.

**Electronic supplementary material:**

The online version of this article (doi:10.1186/s12879-017-2659-3) contains supplementary material, which is available to authorized users.

## Background

The 2015 Ebola outbreak in West Africa was an infectious disease tragedy of epic proportions that drew attention to the daily occupational risks faced by health workers (HWs). One study estimated that during the outbreak in Liberia, 0·11% of the general population died from Ebola compared to 8·07% of the country’s doctors, nurses and midwives [1]. While Ebola dominated the headlines, HWs continue to quietly die from tuberculosis (TB) in numbers far greater than those seen with less common communicable diseases such as Ebola. TB has become a “burgeoning global health crisis” with the emergence of drug-resistant tuberculosis [2] and when coupled with the ongoing struggle to control human immunodeficiency virus (HIV) [3]. Further compounding the TB and HIV epidemics is the critical shortage of HWs globally and especially in Africa [4]. Recent attention to high rates of TB among HWs [5, 6] as well as hospital-based outbreaks of multidrug and extensively drug-resistant TB among patients and workers [3, 7] have led to increased concern about the risk of *Mycobacterium tuberculosis* transmission in healthcare settings. Several studies have confirmed that TB is a significant occupational risk among HWs in low-and middle-income countries [8] and it is estimated that the incidence of TB among HWs in high burden countries (>100 cases/100,000 population) is 8·4% greater (95% CI 2·7%-14·0%) than the general population [9], yet this high-risk population has not been the focus of systematic research. The issue of TB in HWs in low-income countries was highlighted by articles published in the Bulletin of the World Health Organization almost 20 years ago [10, 11], but little has been done to obtain rigorous estimates of the true burden of disease among HWs in regions where TB continues to flourish.

Most estimates of TB disease among HWs in high incidence regions are based on the results of occupational health record reviews thereby excluding HWs diagnosed and treated outside their workplace [5, 6]. Some studies rely on self-reporting of TB status in a climate of HIV and TB associated stigma that predisposes to non-disclosure [7, 12]. A recent study in the province of KwaZulu-Natal of South Africa conducted a retrospective review of TB registers at occupational health clinics in 11 hospitals and four community health centres. The authors concluded that under-reporting of TB among HWs likely masked the true high incidence in this group [13].

Although it is well established that HWs in high burden countries are at high-risk of exposure to TB at work [8, 9, 14], the true incidence rate and burden of TB disease among HWs in South Africa and other low and middle income countries remains unclear. Furthermore, previous methodologies utilized to generate current estimates of TB among HWs suffer from important limitations. To our knowledge, this is the first study in a low/middle-income, high TB burden country to link confirmed cases of TB disease to healthcare human resource records thereby addressing the limitations of self-reporting associated with previous estimates. Other determinants affecting exposure and outcomes among this population are also poorly understood. This is particularly problematic as the lack of good data precludes the prioritization for resource allocation and evaluation of prevention strategies. This study presents incidence rate ratios (IRR) of TB disease among HWs in the Free State province of South Africa from 2002 to 2012. Demographic and clinical characteristics were compared between HWs and the general population with TB. This study also explores the effect of occupational variables on risk of TB among HWs.

## Methods

### Study design and participants

This is a historical prospective cohort study and probabilistic record linkage between the South African national human resource (HR) database called PERSAL and the national TB registry called ETR.Net. These registries do not share a unique identification number.

HWs were defined as “all people engaged in the promotion, protection or improvement of the health of the population” [15]. This definition was not limited to those who provide direct patient care, but was also extended to all who work in a healthcare facility such as cleaners, porters, security personnel, etc. All employees of the Free State Department of Health from 2002 to 2012 who were employed by the health department for at least one month were eligible for inclusion. HWs at all facility levels were included (local clinics, primary, secondary and tertiary hospitals and non-clinical settings).

### Outcomes

Workers with laboratory-confirmed *M. tuberculosis* (including pulmonary, extra-pulmonary, disseminated, miliary and TB meningitis) [16] as identified in ETR.Net were eligible for inclusion in the linkage. HWs with confirmed and documented reactivation of TB were included in the linkage, but were only included in the calculations of the incidence of TB, if the date of diagnosis was after the date of employment. Age, sex, race, HIV status, occupation, facility type, duration of employment, diagnosis type (new, relapse/re-treatment), disease classification (pulmonary, extra-pulmonary, both), outcome (cured/completed, defaulted/failed, transferred/moved, died, unknown) and TB drug sensitivity (multidrug-resistant tuberculosis (MDR-TB) yes, MDR-TB no) were included as covariates. The total number of TB patients in the Free State (general population and HWs) was calculated for each year from ETR.Net. The total number of HWs employed in Free State was recorded from PERSAL and average Full-Time Equivalent (FTE) were calculated for each year (2002–2012).

### Procedures

**Fig. 1:**
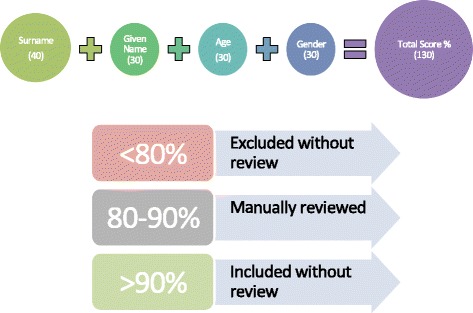
Overview of record linkage weighting and score cut-offs

A probabilistic record linkage was performed to estimate the probability that a PERSAL and ETR.Net record refers to the same person. Raw data were acquired and imported into Microsoft SQL Server 2008 using a custom application. A matching algorithm was written as a custom application using the programming language C#. Following the theories presented by Newcombe [17], variables were assigned a linkage weight according to their reliability and discriminatory power. Based on these parameters, the total weight (or “percentage score”) was derived by summing the separate field comparisons across all fields. A total score was calculated as the sum of surname (40), given name (30), age/date of birth (30), and gender (30) scores where the maximum possible total score was 130 (Fig. 1 and also see Additional file 1 for further details).

Any final percentage scores less than 70% (91 out of 130) were filtered out and were not included in the final dataset. Scores greater than 90% (117 out of 130) were included without manual review. Scores between 80 and 90% were reviewed manually (Fig. 1). Decision rules for manual matching, as described in detail in Table 1, were developed and were employed by two reviewers using a customized web-based tool. Finally, all accepted possible matches were re-assessed by a second reviewer using the same decision rules.

**Table 1:** Manual matching decision rules

Included	Not Included
If difference between birthdates greater than 1 with all other variables an exact match	If only first initial (full first/given name not available) (even if other variables match)
If month and day of birthdate were reversed	If difference between birthdates/year was greater than 2
If one digit of day or month of birth were reversed	If difference between birthdates/year was greater than 1 with at least 1 other variable that was not an exact match
If there was an obvious typo or spelling mistake	If surname was very common (e.g. Mofokeng) and other variables were not perfect matches
If the surnames differed but one could be assumed to be a nickname of the other	If date of birth was different by one or two days
If the given names differed but one could be assumed to be a nickname of the other	If the Surname, Given Name/s and year of birth match perfectly but month and day of birth are different
If all other variables matched perfectly but sex differed	
If the Surname, Given Name/s and year of birth match but month/day in birthdate are missing	
If surname sounds the same but is spelled differently and at least one of the given names and year of birth match	

### Statistical analysis

The total number of TB cases among HWs in Free State was tabulated for each year (2002–2012). Descriptive statistics were utilized to show demographic and clinical characteristics of HWs and the general population with TB in the province.

Person-years at risk for TB for HWs were estimated by assigning a full-time equivalent (FTE) score to each HW. For example, if a HW worked part-time in 2008, they would contribute 0.5 person-years for that year. For each individual, their FTE were summed over the 10 years of the study to generate their individual person-time at risk. Average FTE was then calculated for each year to generate denominators for subsequent calculations.

The number of observed cases of TB among HWs and person years at risk were identified (from HR database PERSAL) for each year (2009–2012). Expected numbers of cases for each year were calculated by multiplying the number of person years at risk each year by the corresponding national TB incidence rate in the general adult population. To calculate the IRR, observed numbers of cases of TB among HWs were divided by the expected numbers in the general adult population for each year.

Poisson regression was used to model the association between facility type, occupation and duration of employment and the rate of TB, with the relative risk being a measure of this association. Birth year, race and sex were entered as independent variables in the multivariate regression to obtain adjusted effects. A random effect for hospital was included producing a generalized mixed-effects regression to account for the fact that HWs are naturally clustered by facility.

To explore the impact of utilizing alternate cut-off scores, IRR were calculated using the number of observed cases from cut-off scores of 80%, 85% and 95% in comparison the 90% presented here. Finally, a sub-set of 390 possible matches that scored within the 90–100% range and a sub-set of 411 possible matches that scored less than 70% were examined manually to validate the computer algorithm and the selection of 70% and 90% as the lower and upper cut-off points.

## Results

**Fig. 2:**
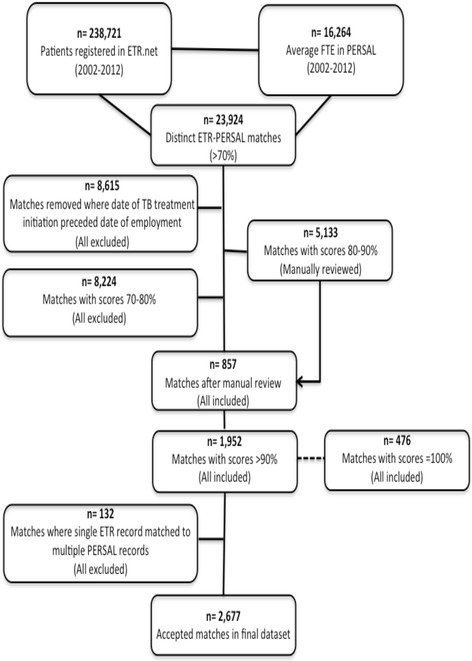
Free State Province ETR.Net- PERSAL record linkage process

A flow chart of the 23,924 partial ETR.Net-PERSAL matches and the procedures employed to obtain the final study population is presented in Fig. 2. Overall, there were 231,834 people diagnosed with TB in Free State from 2002 to 2012. There were 32,039 HWs employed by 258 facilities during this timeframe. During these 11 years, 2677 cases of TB were diagnosed among HWs but only 1280 TB cases were expected. The overall TB incidence rate in HWs during the study period was 1496·32 per 100,000 compared to an incidence rate of 719·37 per 100,000 in the general population during the same time period (Table 2).

**Table 2:** Incidence rate ratios for TB among HWs by year (2002–2012)

Year	HCW Person Years (FTE) ^a^	Observed Cases	Expected Cases	Incidence in General Population (per 100,000) ^b^	Incidence in Healthcare Workers (per 100,000)	Incidence Rate Ratio (95% CI)	*P*-Value ^c^
2002	13,473	80	34	248·5	593·8	2·35 (1·88–2·91)	<0·001
2003	13,964	198	77	552·2	1417·9	2·57 (2·23–2·95)	<0·001
2004	15,101	255	98	648·2	1688·6	2·60 (2·30–2·94)	<0·001
2005	16,448	365	117	708·1	2219·1	3·12 (2·81–3·45)	<0·001
2006	16,391	362	129	787·6	2208·5	2·81 (2·53–3·11)	<0·001
2007	16,641	371	136	815·1	2229·4	2·73 (2·46–3·02)	<0·001
2008	16,722	326	149	891·6	1949·5	2·19 (1·96–2·44)	<0·001
2009	16,404	157	138	842·1	957·1	1·39 (0·97–1·33)	0·106
2010	16,298	203	137	840·0	1245·6	1·48 (1·29–1·70)	<0·001
2011	17,973	191	148	822·7	1062·7	1·29 (1·12–1·49)	<0·001
2012	19,491	169	148	757.0	867·1	1·14 (0·98–1·32)	0·084
Overall TB incidence among HWs for study period					1496·32		

^a^FTE = Full Time Equivalent

^b^As reported by the South African National Department of Health (from ETR.Net) and Statistics South Africa

^c^Chi-Square Test

**Fig. 3:**
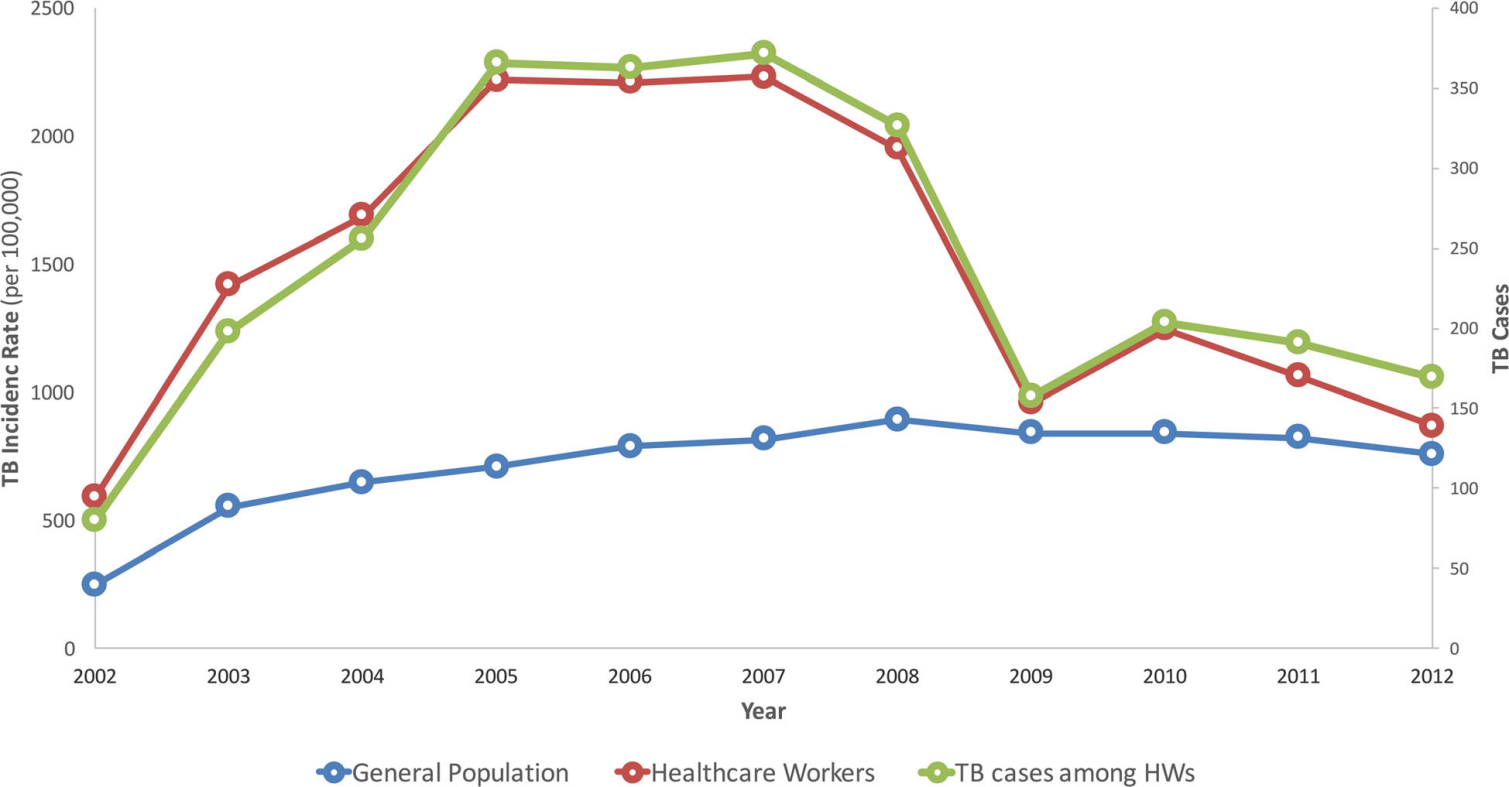
TB incidence rate and TB cases among HWs and the general population (2002–2012)

The number of observed cases of TB among HWs was greater than the number of expected cases for every year during the study period (Table 2). The number of observed cases among HWs ranged from 80 in 2002 to 371 in 2007. The number of TB cases diagnosed among HWs between 2002 and 2012 followed a similar trend over time when compared to the general population (Fig. 3) however, the number of TB cases among HWs was much higher than the general population between 2005 and 2008 and then decreased drastically in 2009. IRR ranged from 1·14 (95% CI: 0·98 to 1·32) in 2012 to 3·12 in 2005 (95% CI: 2·81 to 3·45).

As shown in Table 3, most (*n* = 1989, 74·3%) HWs that were diagnosed with TB from 2002 to 2012 were aged between 30 and 49 years old at the time of diagnosis. There were slightly more females (*n* = 1574, 58·8%) than males and the majority were African/Black (*n* = 2546, 95·1%). About half (*n* = 1551, 57·9%) were employed in a hospital, while 882 (32·9%) worked in a clinic and 244 (9·2%) were employed in “other” settings such as the provincial department of health or central laundry facilities. Most HWs with TB were nurses (*n* = 1113, 41·6%), 767 (28·7%) were support staff such as maintenance workers, laundry workers, food service workers, security personnel, cleaners and porters, 282 (10·5%) were physicians and surgeons, and 407 (15·2%) were administrative staff. There were 108 (4·2%) allied health professionals (physical therapists, audiologists, technologist/technicians, pharmacists, social workers and dieticians) with TB during the study period. Most HWs with TB did not know their HIV status or did not disclose it (*n* = 2035, 75·6%) but 498 were known to be HIV positive (18·6%). Pulmonary TB was diagnosed in 2039 (76·2%) cases. There were 560 cases (20·9%) of extra-pulmonary TB and 78 (2·9%) were classified as having both pulmonary and extra-pulmonary disease. Most (*n* = 2149, 80·3%) were newly diagnosed cases with the rest being relapses or re-treatment cases. There were only 18 documented cases in ten years that were classified as being multiple-drug resistant TB (MDR-TB) among HWs. The majority (*n* = 1742, 65·1%) of HWs completed their course of treatment and were classified as “cured” while 306 (11·3%) died. One hundred and thirty-six (5.1%) defaulted or failed treatment, 405 (15·1%) transferred or moved out of province and the outcome was unknown for 90 HWs (3·4%).

**Table 3:** Demographic and clinical characteristics of HWs and the general population with TB in Free State, South Africa

Variable	Health Workers (*N* = 2677) Frequency (%)	General Population (*N* = 229,157) Frequency (%)
Age (at diagnosis)		
< 19	0 (0·0)	4714 (2·1)
20–29	205 (7·7)	51,409 (22·4)
30–39	992 (37·1)	76,463 (33·3)
40–49	997 (37·2)	58,223 (25·4)
50–59	429 (16·0)	27,598 (12·0)
60+	54 (2·0)	10,750 (4·7)
Sex		
Male	1103 (41·2)	98,872 (43·1)
Female	1574 (58·8)	130,285 (56·0)
Race		
African	2546 (95·1)	−
White	70 (2·6)	−
Coloured	59 (2·2)	−
Indian	2 (0·08)	−
HIV Status		
HIV+	498 (18·6)	71,778 (31·3)
HIV-	154 (5·8)	22,420 (9·8)
Unknown	2025 (75·6)	98,959 (43·2)
Facility Type		
Hospital	1551 (57·9)	−
Clinic	88 (32·9)	−
Other	244 (9·2)	−
Occupation		
Doctor/Surgeon	282 (10·5)	−
Nurse	1113 (41·6)	−
Allied Health Professional	108 (4·2)	−
Administrative/Clerical	407 (15·2)	−
Support Services	767 (28·7)	−
Duration of Employment		
1–5 years	529 (20·0)	−
6–10 years	359 (13·4)	−
11–15 years	536 (20·0)	−
16–20 years	498 (18·6)	−
20+ years	461 (17·2)	−
Disease Classification		
Pulmonary	2039 (76·2)	179,579 (78·4)
Extra-pulmonary	560 (20·9)	44,952 (19·6)
Both	78 (2·9)	4626 (2·0)
Diagnosis Type		
New	2149 (80·3)	186,679 (81·5)
Relapse/Re-treatment	528 (19·7)	42,478 (18·5)
MDR-TB (pre-treatment)		
Yes	18 (0·7)	1430 (0·6)
No	2659 (99·3)	227,727 (99·4)
Outcome		
Cured/Completed	1742 (65·1)	134,857 (58·8)
Defaulted/Failed	136 (5·1)	14,783 (6·5)
Transferred/Moved	405 (15·1)	38,585 (16·8)
Died	306 (11·3)	29,156 (12·7)
Unknown	90 (3·4)	11,776 (5·1)

*Physicians and Surgeon = Surgeon, Radiologist, Anaesthesiologist, Other Physicians, Medical Registrar; Nurse = Professional nurse, Assistant or auxiliary Nurse, Staff Nurse; Allied Health Professional = Therapist (i.e. audiologist), Technologist/technician, Pharmacist, Social Worker; Administrative/Clerical = Manager/administrator, Clerk, General Assistant; Support Staff = Maintenance Worker, Laundry Worker, Food Service Worker, Security, Cleaner, Porter

Table 4 shows the unadjusted and adjusted relative risk estimates from the mixed-effects Poisson regression model. The final adjusted model included birth year, race, sex, occupation and duration of employment. The risk of TB disease was greatest among HWs born from 1960 to 1969 (RR = 7·29, 95% CI: 5·48 to 9·72) when compared to those who were born in the 1980’s. Black/African HWs had a greater than 5-fold increased risk of TB when compared to their white colleagues (RR = 5·30, 95% CI: 3·90 to 7·20). Similarly, coloured HWs (people of mixed ethnic origin) had an almost 3-fold increased risk of TB (RR = 2·90, 95% CI: 1·87 to 4·50). The risk of TB was greater among male compared to female HWs (RR = 1·51, 95% CI: 1·38 to 1·64). In the unadjusted analysis, TB risk was greater among support staff (RR = 2·36, 95% CI: 1·79 to 3·11), nursing staff (RR = 2·07, 95% CI: 1·58 to 2·71) and administrative staff (RR = 1·74, 95% CI: 1·29 to 2·32) when compared to allied health professionals. These estimates attenuated in the adjusted model and were no longer statistically significant. The risk of TB was also slightly higher among doctors and surgeons in the unadjusted analysis, but this finding was not statistically significant (RR = 0·85, 95% CI: 0·62 to 1·15). The RR estimates for all occupation categories attenuated in the adjusted model and were no longer statistically significant. HWs who had worked in the healthcare sector for less than 20 years had a greater risk of TB compared to those who had been employed for more than 20 years. In particular, HWs who were employed for 11–15 years had a more than 3-fold increased risk of TB (RR = 3·60, 95% CI: 2·97 to 4·37). Facility type was not associated with increased risk of TB.

**Table 4:** Relative risk estimates from mixed-effects poisson regression model of HWs with TB and those without TB (*N* = 32,039)

	Unadjusted	*P*-Value	Adjusted	*P*-Value
	RR (95% CI)		RR (95% CI)	
Birth Year				
1980–1989	1·00		1·00	
1970–1979	4·28 (3·24 to 5·65)	<0·0001	3·84 (2·89 to 5·09)	<0·0001
1960–1969	7·95 (6·06 to 10·42)	<0·0001	7·29 (5·48 to 9·72)	<0·0001
1950–1959	6·10 (4·59 to 8·10)	<0·0001	7·11 (5·22 to 9·69)	<0·0001
1940–1949	3·43 (2·43 to 4·85)	<0·0001	4·03 (2·78 to 5·83)	<0·0001
1930–1939	1·79 (0·16 to 20·84)	0·99	1·39 (0·13 to 15·13)	0·99
Race				
White	1·00		1·00	
African	6·74 (4·98 to 9·11)	<0·0001	5·30 (3·90 to 7·20)	<0·0001
Coloured	3·59 (2·32 to 5·56)	<0·0001	2·90 (1·87 to 4·50)	<0·0001
Asian	1·15 (0·18 to 7·02)	0·99	1·05 (0·18 to 6·07)	0·99
Sex				
Female	1·00		1·00	
Male	1·41 (1·31 to 1·53)	<0·0001	1·51 (1·38 to 1·64)	<0·0001
Facility Type				
Non-Clinical	1·00		−	−
Hospital	2·04 (0·79 to 5·26)	0·19	−	−
Clinic	2·47 (0·67 to 9·12)	0·24	−	−
Occupation				
Allied Health Professional	1·00		1·00	
Doctor/Surgeon	1·13 (0·83 to 1·53)	0·84	0·85 (0·62 to 1·15)	0·60
Nurse	2·07 (1·58 to 2·71)	<0·0001	1·24 (0·93 to 1·63)	0·25
Administrative/Clerical	1·74 (1·29 to 2·32)	<0·0001	1·13 (0·84 to 1·51)	0·82
Support Staff	2·36 (1·79 to 3·11)	<0·0001	1·28 (0·96 to 1·70)	0·14
Duration of Employment (yrs)				
20+	1·00		1·00	
16–20	2·05 (1·71 to 2·47)	<0·0001	1·97 (1·63 to 2·39)	<0·0001
11–15	3·36 (2·80 to 4·04)	<0·0001	3·60 (2·97 to 4·37)	<0·0001
6–10	1·21 (0·99 to 1·48)	0·09	1·72 (1·38 to 2·14)	<0·0001
1–5	1·06 (0·89 to 1·28)	0·97	1·92 (1·56 to 2·37)	<0·0001
< 1	2·74 (2·24 to 3·53)	0·03	1·60 (1·44 to 1·82)	<0·0001

**Fig. 4:**
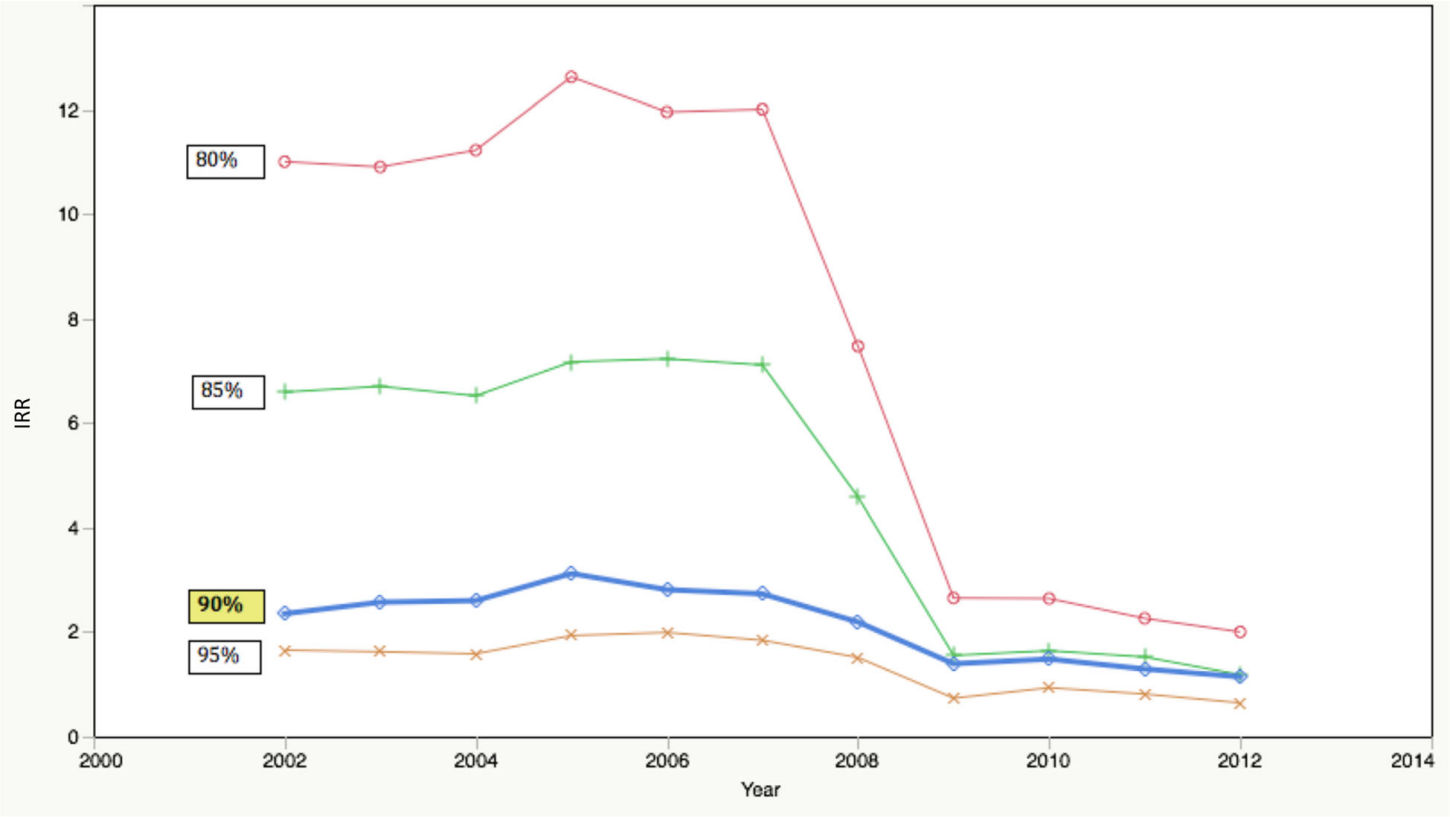
Incidence rate ratio by year and percentage cut-off score

Figure 4 provides a visual depiction of the IRR over time for cut-off scores of 80%, 85%, 90% and 95%. With an 80% cut-off point, IRR ranged from 12·62 in 2005 to 2·00 in 2012. With an 85% cut-off point, the range was from 7·23 in 2006 to 1·52 in 2011. With a 95% cut-off point, IRR ranged from 1·99 in 2006 to 0·64 in 2012. Manual matching of the 411 records that scored <70%, resulted in only 2 that were deemed to be false negatives that should have been included in the linked dataset and 409 that were appropriately discarded. Similarly, manual matching of the 390 records that scored >90%, 383 were true positive matches and that only 7 were false positives that should have been excluded.

## Discussion

These findings confirm that HWs in Free State, South Africa have higher rates of TB than the general population. Although the rates were higher than the general population in all study years, the excess of cases was particularly high from 2002 to 2008 and highest in 2005. For this year, there was an alarming 312% more cases of TB among HWs than expected meaning that the incidence of TB was more than 3-fold greater among HWs than the general population in this year.

We observed a dramatic drop in HW TB rates around 2009. It is possible that this could in part be explained by the implementation of ‘The Draft National Infection Prevention And Control Policy For TB, MDRTB And XDRTB’ and the ‘Tuberculosis Strategic Plan For South Africa, 2007-2011’ across the country in 2007. These two policy documents had implications for TB infection control in health care settings. We were not able to identify any formal changes to the reporting systems during the study period. It is also possible that the drop in TB rates could be explained by the aggressive role out of a free antiretroviral treatment program in the country in 2004. It is estimated that there were 919,923 HIV patients enrolled in the public program by November 2009- a drastic increase from only 32,895 in January 2005 [18]. It is also possible that the case definitions used in the ETR.Net system were changed. For example, if they changed the way they entered re-infections for the same person, there would have been a drop in incidence. Further investigations are necessary to fully explore the cause of the drop in incidence rates in 2009.

Our estimates of TB among HWs are consistent with other reports from the region including a study by O’Donnell and colleagues from South Africa estimated rates of MDR and extensively drug-resistant tuberculosis (XDR-TB) related hospital admissions [7] and a retrospective record review in one hospital in Kenya to document TB case notification rates among hospital staff [19]. The results presented here show that almost 30,000 people died from TB in Free State during the study period. More than 300 of those who died were HWs. This loss of skilled personnel is a huge detriment to a health system that is over-burdened by the TB/HIV syndemic and where health human resource shortages are common. We found that there were more TB patients in the 60+ age category and in the age categories <29 in the general population group than in the HW group. This is likely due to the fact that many HWs retire in their sixties and may still be completing their education and training in their twenties and therefore are not yet employed. WHO estimates that 61% of TB patients in South Africa are co-infected with HIV [20]. These findings suggest that only 31·3% of the general population (non-HWs) were known to be HIV positive. It was also interesting to note that the rate of TB patients who were known to be HIV positive was still much higher in the general population group when compared to the HW group. This is because the HIV status was unknown for the majority of HWs with TB (75·6%) suggesting that HWs in Free State are either not receiving adequate access to HIV counselling and testing or that they are afraid to disclose their status.

Similar to the study from Kenya [19], HWs in Free State had sub-optimal cure rates. HWs must therefore receive early diagnosis and treatment for TB in addition to improved infection prevention and control efforts [21] in accordance with international guidelines [22]. A survey administered to medical doctors diagnosed with TB in South Africa found that a prompt diagnosis within 7 days was only made in 20% of participants and 95% of respondents expressed concerns regarding a lack of IC in the workplace and negative attitudes of senior administrators and colleagues [23]. HWs should also be screened regularly for TB by programs that are free, confidential and available in the workplace [24]. These results also show that occupation and facility type are not as strongly associated with increased incidence of TB among HWs as expected. This suggests that all HWs who work in hospitals, clinics and even administrative settings are at risk of exposure to TB in the workplace and that there should be greater effort to include non-clinical personnel in TB infection control education and training. These findings also show that health workers with less healthcare sector work experience (employed less than 20 years) were more likely to have TB than those who were employed more than 20 years. Interestingly, health workers who were employed for 11–15 years had the highest risk of TB. This could perhaps be due to what is known as the “healthy worker survivor effect.” This is the tendency for the least healthy workers to leave the active workforce. Furthermore, health workers who worked more than 20 years may have also been able to better protect themselves, may have had work tasks with less exposure, and may have had fewer concomitant risk factors.

Although this probabilistic record linkage study is the first in the region to objectively estimate TB incidence among HWs, it does have several limitations. First, the quality of the data in ETR.Net is variable as the system relies on input from paper forms collected by nurses at each facility. Furthermore, the information contained in ETR.Net did not allow us to distinguish between relapse and retreatment cases. We recognize that the major risk factors for relapse include inadequate therapy due to irregularity, high disease burden in the population, inadequate duration of therapy and underlying drug resistance. Recurrence of disease due to true relapse would ideally be distinguished from reinfection. Occupational cohort studies are vulnerable to several biases such as misclassification bias. Misclassification bias on exposure is not likely in this study however misclassification of the outcome (TB status) is possible. The ETR.Net registry does not necessarily contain all records of patients diagnosed with TB and HWs in particular may be less likely to report their disease. It is therefore possible that the estimates of TB among HWs are under-reported here. Despite these limitations, the results of the sensitivity analysis shown in Fig. 2 illustrate that 90% was a reasonable cut-point to accept all matches. The quality of the matches decreased dramatically at 85% as evidenced by the large jump in IRR. With all cut-off scores (80%, 85%, 90% and 95%) there is a noticeable drop in IRR in 2008–2009 as discussed previously.

South Africa has adequate policies in place for the protection of HWs from TB and other workplace conditions [25–28] yet this study illustrates the urgent need for the implementation of these policies, in particular TB infection prevention and control measures and occupational health and safety practices [29]. There is a need for better workplace as well as workforce surveillance, with prompt follow-up of cases of HWs with TB to ensure that all infection control measures are being followed in areas in which staff that contracted TB worked. HWs work in stressful environments where they are at high risk of exposure to infectious diseases such as hepatitis, HIV, TB and even Ebola. Many HWs who are diagnosed with TB, report feeling stigmatized [30] and unsupported in their journey back to health [30]. The findings presented here re-affirm the urgent call for action to protect the healthcare workforce.

## Conclusions

HWs in Free State, South Africa have higher rates of TB than the general population. HWs are the backbone of health systems worldwide and this study reinforces that we are not doing enough to protect them. Additional efforts must be made to protect this high-risk, high-value population by implementing effective infection control measures and providing timely TB screening, diagnosis, treatment and support.

### What is already known about this topic

Several small studies, based on occupational health clinic records or hospital admission data, have suggested that healthcare workers are at increased risk for acquiring tuberculosis.

### What new knowledge this study contributes

This historical prospective cohort study is the first record linkage which documents that healthcare workers in the Free State province of South Africa have an up to three-fold risk of TB disease compared to the general population.

## Additional file

Additional file 1: Record linkage technical appendix. (DOCX 118 kb)

## Abbreviations

CI: confidence interval
FTE: full time equivalent
HIV: human immunodeficiency virus
HR: human resources
HWs: health workers
IRR: incidence rate ratio
MDR-TB: multidrug-resistant tuberculosis
RR: relative risk
TB: tuberculosis
XDR-TB: extensively drug-resistant tuberculosis
