# Supplementary material for: The neglected burden of tuberculosis disease among health workers: a decade-long cohort study in South Africa
Source: BMC Infect Dis. 2017 Aug 7;17:547. doi: 10.1186/s12879-017-2659-3 (PMC5547542; doi:10.1186/s12879-017-2659-3)
Supplement: Additional file 1: — Record linkage technical appendix. (DOCX 118 kb) [file 12879_2017_2659_MOESM1_ESM.docx]

**Record linkage technical appendix**

Raw data were acquired (PERSAL and ETR databases) from 2002-2012. Both databases were in .CSV format. Data were imported into Microsoft SQL Server 2008 and a custom application was used to perform this task. A simple check for data integrity was performed (on ETR) and any rows that did not pass were removed. Any rows where “last name” was empty were removed and any rows where “facility” was missing or incomplete were removed. This removed 16 rows (16/ 239,204 total rows in ETR= 0.00668%) of data from ETR. A “Region” field was created in both ETR and PERSAL databases using a compiled list of hospital/clinics. In PERSAL, name matching was performed to determine the region of approximately 80% of the entries. Of the remaining entries, the programmer was able to match about half of them manually to a region. In these cases, the name match had failed due to either typos or abbreviated names. ETR had much fewer facilities listed than PERSAL, so 100% of entries were assigned a region. The rationale for including region was that an individual is unlikely to receive TB treatment in a region that is very far from their workplace/home. However, it is possible that individuals live near regional borders and may live/work in Clocolan for example (Thabo Mofutsanyana region) and drive to Landybrand (Motheo region) for TB treatment. In spite of this limitation, region was deemed to be additional useful information and was included as an unweighted variable in the manual matching.

The linked dataset was then kept in an encrypted password-protected file on a UBC server. Data cleaning, development and refinement of the linkage algorithm and protocols and manual review of records were conducted at the University of British Columbia in Vancouver, Canada between April 2013- December 2014. Reports from Statistics South Africa and the Human Sciences Research Council were downloaded directly from the websites as CSV files. Data analysis was conducted between October 2014- September 2015.

## Matching algorithm

The matching algorithm was written as a custom application with programming language C#. The program accessed two different Microsoft SQL Server database tables (ETR, PERSAL) that were imported and cleaned in the previous steps and created a new matching table.

When the ETR and PERSAL data were imported, Microsoft SQL Server randomly assigned a unique index to each row. These indices were used to reference the ETR and PERSAL rows in the matching table that were generated. This allowed the data to be anonymized after they had been matched; however, if the unique index of the PERSAL ID had been used, it would not have been possible to anonymize the data.

Variables were assigned a linkage weight according to their reliability and discriminatory power. Reliability or *m* probability is the probability that a matching variable agrees given that the comparison pair is in fact a match. Discriminatory power or *u* probability is the probability that a matching variable agrees purely by chance (is a non-match). Based on these parameters, the total weight (or “percentage score”) is derived by summing the separate field comparisons across all fields.

For the record linkage described here, surname was evaluated first. If it was an exact match, it was assigned a perfect score of 40 for surname, and the program proceeded to assess given name. If surname was not an exact match, a coefficient for the inaccuracy of the name was created (“sc”=surname coefficient). Levenstein distance was calculated between the surname as registered in ETR and the surname in PERSAL (“sd”=surname distance). The number of letters in the longest surname in either ETR or PERSAL was then calculated (“sl”=surname length). By definition, sl >= sd. We then say sc = sd / sl, and therefore 0 <= sc <= 1. This means that sc is small when there is little distance between the names matched, and sc is large when they are very different.

Next the program checked if the two names sound the same, based on the double metaphone algorithm. If they do sound the same (eg. Steven and Stephen), 0.05 was subtracted from sc. Only a small weight was placed on the double metaphone algorithm because it tends to be less accurate than the Levenstein distance. Next, the first initials of the surname were compared (eg. **B**aker and **B**arker). If they were the same, 0.1 was subtracted from sc**.** If at this point sc < 0, sc was reset to equal 0. The calculation was then finalized for the score of an imperfect surname match. With 0 <= sc <= 1, with sc closer to 0 when there was a close match, a percentage of a score of a max score of 35 was derived (40 was the score assigned for a perfect match, which is impossible to achieve for an imperfect match.) Therefore, Score = 35 * (1 – sc).

Given name matching was in almost an identical manner to the Surname matching, with the following two exceptions;

1. The score for a perfect match is 30, and the max score for an imperfect match is 25. For imperfect matches, Given Name Score = 25 * (1 – gc).
2. There could be many given names; for example, PERSAL could have the given names “Stephen John” and ETR might only have “Stephen”. The algorithm does not penalize the score if a name is missing from one of the databases. “Stephen” and “Stephen John” would be considered a perfect match for 30 points.

To generate a score for age, the number of days difference, between the birthdates listed in ETR and PERSAL was calculated. If the difference was 0, an age score of 30 was assigned. If the difference was less than 150, the score was 25. If the difference was less than 366, the score was 20. If the difference was less than 500, the score was 10. If the difference was less than 1000, the score was 5. A difference up to 366 could still indicate a perfect match, as the ETR database does not always have a precise date of birth. It does contain the age, and date of treatment, which can be used to reverse calculate a possible birthdate. However, this date could potentially be inaccurate up to 365 days. These cases were flagged for manual matching so that the reviewer was aware.

For sex, a simple comparison of male vs. female was used. If it was the same, sex score was 30; otherwise, it was 0.

Based on these parameters, final percentage scores were assigned to each comparison. A total score was calculated as the sum of surname (maximum possible score=40), given name (30), age (30), and sex (30) scores where the maximum possible score was 130.

Any final percentage score less than 70% (91 out of 130) was filtered out and were not included in the final dataset. Scores greater than 90% (117 out of 130) were included without manual review. Scores between 80-90% were reviewed manually.

### Manual review of partial matches

Decision rules for manual matching were developed and were employed by two reviewers. After the PERSAL-ETR linkage was complete, the link was reversed to determine how many ETR records were linked with multiple PERSAL entries. Finally, all accepted possible matches were re-assessed by a second reviewer using the same decision rules.

The computerized steps in the record linkage described above should have generated all possible matches and matched pairs between the ETR and PERSAL databases. However, this dataset also included many false-positive matches and therefore manual review by a human is necessary to remove as many of these false-positives as possible. A custom web-based tool was used to facilitate manual matching of possible matches in the grey area.

In many cases, a single PERSAL employee was linked to multiple entries in the ETR database, especially if they received multiple TB treatments. If the reviewer determined that the displayed data shows such a case, all such records were included for reasons discussed previously. It is also possible that the algorithm only returned false positives, for a single PERSAL employee, these cases were not included. After clicking “Save”, a new employee with its matches are loaded onto the screen and this process was repeated for all possible matches.
